# Regulatory T Cells Control the Switch From *in situ* to Invasive Breast Cancer

**DOI:** 10.3389/fimmu.2019.01942

**Published:** 2019-08-29

**Authors:** Leandro M. Martinez, Valentina Robila, Nicholas M. Clark, Wei Du, Michael O. Idowu, Melanie R. Rutkowski, Paula D. Bos

**Affiliations:** ^1^Department of Pathology, Virginia Commonwealth University School of Medicine, Richmond, VA, United States; ^2^Anatomic Pathology Service, Department of Pathology, Virginia Commonwealth University School of Medicine, Richmond, VA, United States; ^3^Integrative Life Sciences Doctoral Program, Virginia Commonwealth University, Richmond, VA, United States; ^4^Microbiology, Immunology, and Cancer Biology, University of Virginia, Charlottesville, VA, United States; ^5^Massey Cancer Center, Virginia Commonwealth University School of Medicine, Richmond, VA, United States

**Keywords:** regulatory T cells, non-invasive carcinoma, early stage breast cancer, immunosurveillance, immunotherapy

## Abstract

Ductal carcinoma *in situ* (DCIS) is a non-obligate precursor of breast cancer, and it only progresses to invasive breast cancer in around 40% of patients. While immune infiltrates have been observed in these early cancer lesions, their potential prognostic value is still unclear. Regulatory T (Treg) cells accumulate in advanced breast cancers, and predict poor outcome. We have shown before that ablation of Treg cells in established tumors leads to significant decrease in primary and metastatic tumor burden. In this work, we sought to investigate Treg cell function in the progression from non-invasive to invasive breast cancer lesions. To this end, we used the murine mammary tumor virus polyoma middle T (MMTV-PyMT) murine model of spontaneous, stage-wise breast carcinogenesis crossed to Foxp3^*DTR*^ knock in mice, allowing Treg cell ablation by administration of diphtheria toxin. Transient targeting of Treg cells at the *in situ* carcinoma stage resulted in a significant increase in the number of tumor-bearing mammary glands and size of growing tumors compared with control mice. Whole mammary gland mounts and histological examination confirmed larger emergent tumor area in Treg cell-ablated mice, and revealed that these tumors were characterized by a more advanced tumor staging, with presence of early invasion, increased desmoplasia and collagen deposition. Furthermore, Treg cell ablation increased the percentage of cancer stem/progenitor cells in the mammary compartment. Interestingly, Treg cell ablation resulted in increased inflammatory cytokines IL-4 and IL-5 with a concomitant reduction in classically activated tumor associated macrophages. This TH2-biased immune regulatory mammary inflammation was consistent with the enhancement in tumor promotion that we observed. Overall, our study demonstrates that Treg cells oppose breast cancer progression at early stages, raising a cautionary note regarding the consideration of immune intervention targeted at boosting immune responses for DCIS.

## Introduction

While death from breast cancer has slowly declined in the past few years, mammographic screening has led to a dramatic increase in the detection of pre-invasive breast lesions in women (1–3). This paradoxical observation can be explained by the fact that only a low percentage of early breast disease progresses to invasive, metastatic carcinomas. Ductal carcinoma *in situ* (DCIS) is a heterogenous group of neoplastic lesions confined to the breast ducts, and can remain indolent for life in up to 60% of cases (2). Patients diagnosed with DCIS undergo breast-conserving therapy or mastectomy, frequently accompanied by radiotherapy and in some cases, hormonal therapy (4). Thus far, there are no reliable parameters to distinguish those cases that will progress, resulting in significant overtreatment (5). Furthermore, our sparse understanding of the mechanisms leading to the transition from pre-invasive to invasive cancer deprives patients from targeted therapies that could improve outcomes (6, 7). Therefore, identifying cellular or molecular drivers of early tumor invasion may lead to the identification of biomarkers that can reduce the overtreatment in low-risk invasive breast cancer patients, or actionable targets that enable early management of the disease (5).

Evidence of tumor-infiltrating lymphocytes paralleling disease progression suggests that the interactions of immune cells and tumor cells are important for tumor evolution (8). T cell presence is a positive indicator of good prognosis, suggesting an active involvement in immunosurveillance (8). On the other hand, suppressive Foxp3^+^ regulatory T (Treg) cells, which represent a significant proportion of the CD4^+^ population in tumors, have been shown to increase with tumor stage and correlate with poor prognosis in invasive carcinomas (9). We have demonstrated that ablation of Treg cells in advanced primary tumors induces a strong anti-tumor response, which is dependent on CD4^+^ T cells and IFNγ (10). However, the role of Treg cells during the initial stages of breast cancer tumorigenesis remains obscure. In the present work, we addressed the effect of transiently ablating Treg cells during the non-invasive stage, using a spontaneous model of breast carcinogenesis driven by expression of the polyoma middle T oncogene from the murine mammary tumor virus LTR (MMTV-PyMT). Our results indicate that transient Treg cell ablation in *in situ* breast lesions results in acceleration of progression to invasive carcinoma, suggesting that Treg cell presence may be a positive prognostic indicator for pre-invasive breast cancer.

## Materials and Methods

### Mouse Models

*Foxp3*^*DTR*−*GFP*^ mice were a gift from A. Rudensky (Memorial Sloan Kettering Cancer Center, New York, NY). C57BL/6 *MMTV-PyMT* mice were generously provided by M.O. Li (Memorial Sloan Kettering Cancer Center, New York, NY). All animal protocols were reviewed and approved by VCU Institutional Animal Care and Use Committee (IACUC #AD10001219).

### Primary Tumor Growth Evaluation

Primary tumor incidence and growth was monitored weekly by palpation of all mammary glands, and caliper measurements of the length (L) and width (W) of each tumor. Individual tumor volume was calculated using the formula πLW^2^/6. For Kaplan-Meier analysis of disease-free survival, a mouse was no longer considered disease-free when the first tumor reached a diameter of 2 mm.

### Histology

We restricted all histological analysis to the fourth pair of mammary glands. Whole mounts were obtained as described Rasmussen et al. (11). Briefly, mammary glands were resected and spread onto a glass slide, fixed in Carnoy's fixative for 4 h at room temperature, and progressively hydrated. Glands were then rinsed in tap water, stained in carmin alum overnight, dehydrated, and cleared in xylene. Glands were mounted with Permount and scanned using an Olympus BX51 + CAST2 Stereology System microscope. Tumor area was calculated as a percentage of total area using Image J software. Whole mounts were subsequently embedded in paraffin and sectioned at 5–7 μm thickness. Hematoxylin and eosin (H&E) staining was carried out following standard protocols. All histological analysis and scoring were performed by a blinded expert breast pathologist. Tumor staging was scored as percentage of each stage out of the whole tumor area, as described by Lin et al. (12). Histological characteristics such as intra-tumor inflammatory infiltrate, desmoplasia, and collagen deposition were scored using a 0–3 scale as: absent (0); scanty (1); moderate (2); or extensive (3). Collagen deposition was evaluated by Masson Trichrome stain following specific manufacturing recommendations (NavaUltra Masson Trichrome Stain Kit, cat IW-3006). For alpha smooth muscle actin (α-sma) immunofluorescent staining, sections were deparaffinized, and rehydrated. Antigen retrieval was performed by incubation in citrate buffer (0.01 M, pH 6) at 60°C for 30 min. Tissues were then blocked with 1% bovine serum albumin (BSA) in PBS for 1 h, and incubated with eFluor 660 conjugated primary antibodies anti-α-sma (1/100, 50-9760-82, eBioscience). Slides were then washed in PBS and mounted with DAPI containing Vectashield medium (H-1500, Vector). All images were acquired on a Microbrightfield-Neurolucida System microscope.

### Flow Cytometry

Efficiency of systemic and local Treg cell ablation over time was evaluated in peripheral blood and mammary gland, respectively, by calculating the frequency of CD4^+^ Foxp3^+^ cells. For all flow cytometric analysis of mammary glands, the whole mammary gland was dissected, and central lymph node removed. Tissues were minced and enzymatically digested using 400 μg/ml Liberase TL (Roche) in a rotary shaker for 30 min at 37°C. Single cells were obtained by filtration through 100 μm cell strainers (Fisherbrand) and centrifugated at 300 × g for 5 min. Cells were incubated for 20 min in FC block (anti-CD16/32, Tonbo) on ice. Cells were then stained for 30 min on ice with specific antibody cocktails diluted in PBS with 0.5% BSA: violet Fluor 450-conjugated Ab anti-CD4 (1/500, 75-0042, TONBO), BUV395-conjugated anti-CD45 (1/1000, 565967, BD Biosciences), eFluor 450-conjugated anti-CD24 (1/500, 75-0242, TONBO), red Fluor 710-conjugated anti-CD44 (1/500, 80-0441-U025, eBioscience), PE-Cy7-conjugated anti-CD49f (1/500, 25-0495-82, eBioscience), APC-conjugated anti-CD29 (1/500, 17-0291-82, eBioscience), FITC-conjugated anti-CD61 (1/500, 11-0611-82, eBioscience), APC-eFluor 780-conjugated anti-CD11b (1/1000, 47-0112-82, eBioscience), PE-Cy7-conjugated anti-Ly-6C (1/500, 25-5932-82, eBioscience), FITC-conjugated anti-Ly-6G (1/500, 127605, Biolegend), PerCP-Cy5.5 -conjugated anti-F4/80 (1/500, 45-4801-82, eBiosciences), redFluor™ 710-conjugated anti-MHC Class II (1/500, 80-5321-U025, TONBO), Alexa Fluor 647-conjugated anti-CD206 (1/500, MCA2235A647T, Serotec). Ghost Violet 510 viability dye (13-0870-T100, TONBO) was used to discriminate live/dead cells. For intracellular staining, cells were permeabilized using the FoxP3/Transcription Factor Staining Buffer Kit (TNB-0607-KIT, TONBO) according to the manufacturer's instructions, and stained using FITC-conjugated anti-FoxP3 antibody (11-5773-82, eBioscience). After staining, cells were washed and fixed in 2% paraformaldehyde. Flow cytometry was carried out using LSRFortessa-X20™ equipment (BD). Data analysis was performed using FlowJo 10.2 software.

### *In vivo* Tumor Initiating Capacity

Single mammary cell suspensions from control and diphtheria toxin (DT)-treated mice were obtained from the mammary gland at 10 weeks of age, as previously described for flow cytometry. Briefly, 325,000 live cells were re-suspended in PBS and mixed at a 1:1 ratio in growth factor reduced Matrigel (BD). Cell suspensions were injected bilaterally into the fourth mammary gland of isoflurane-anesthetized C57BL/6 mice. Primary tumor growth was weekly monitored and tumors were harvested at the humane end-point.

### *In vitro* Mammosphere Assay

Single mammary gland cell suspensions were depleted of hematopoietic cells by incubation with anti-CD45 (70-0451, TONBO), followed by Dynabeads® Sheep anti-Rat IgG according to the manufacturer's instructions (Invitrogen, 11035). Depletion was confirmed by flow cytometry using APC-conjugated anti-CD45.2 (20-0454-U025, TONBO). Mammosphere assay was performed as described by Boyle et al. (13), with a few modifications. Briefly, 2 × 10^4^ freshly isolated CD45^−^ mammary gland cells were seeded in triplicates into 96-well ultra-low attachment plates (Corning Inc.) pre-coated with poly(2-hydroxyethyl methacrylate) (P3932, Sigma) in a 1:1 mixture of DMEM and Ham's F12 medium (Sigma) supplemented with NeuroCult SM1 Neuronal Supplement (05711, StemCell Technologies), 20 ng/ml bFGF (78003.1, StemCell Technologies), 20 ng/ml EGF (78006, StemCell Technologies), 4 μg/ml heparin (07980, StemCell Technologies), penicillin-streptomycin and fungizone. Mammosphere cultures were incubated at 37°C for 7 days. At the end point, mammospheres of at least 40 μm diameter were counted under the microscope at 40X magnification. Digital images were used to calculate mammosphere size using Image J software.

### Cytokine Analysis

Tumors were lysed in buffer containing 50 mM Tris, 150 mM NaCl, 1% NP-40, 1 mM EDTA, and protease inhibitors. Cleared lysates were quantified and extracts bearing 20 μg of total protein were used to quantify specified cytokines using a Luminex bead assay (Millipore), according to the manufacturer's instructions.

### Quantitative PCR Analysis

Frozen mammary glands were pulverized on a dry ice bed, and resuspended in TRIzol reagent (Invitrogen). RNA was extracted following standard protocols and reverse-transcribed using SuperScript III Reverse transcription kit (Invitrogen). Semi-quantitative PCR was performed using an ABI Prism 7900HT instrument (Applied Biosystems) and SybrGreen PCR master mix (Applied Biosystems). The indicated transcripts were assayed using the following primers:

β-actin forward, 5′-AAGGCCAACCGTGAAAAGAT-3′;

β-actin reverse, 5′-GTGGTACGACCAGAGGCATAC-3′;

F4/80 forward, 5′-GGAGGACTTCTCCAAGCCTATT-3′;

F4/80 reverse, 5′-AGGCCTCTCAGACTTCTGCTT-3′;

iNOS forward, 5′-CTTTGCCACGGACGAGAC-3′;

iNOS reverse, 5′-TCATTGTACTCTGAGGGCTGAC-3′;

Arg-1 forward, 5′-GAATCTGCATGGGCAACC-3′;

Arg-1 reverse, 5′-GAATCCTGGTACATCTGGGAAC-3′.

### Statistical Analysis

Statistical analysis was performed with Prism software (GraphPad Software), using parametric and non-parametric tests, as indicated in each figure. Differences were considered statistically significant when *p* < 0.05 (two-tailed).

## Results

### Treg Cell Ablation During the Non-invasive Stage Accelerates Breast Primary Tumor Growth

**Figure 1:**
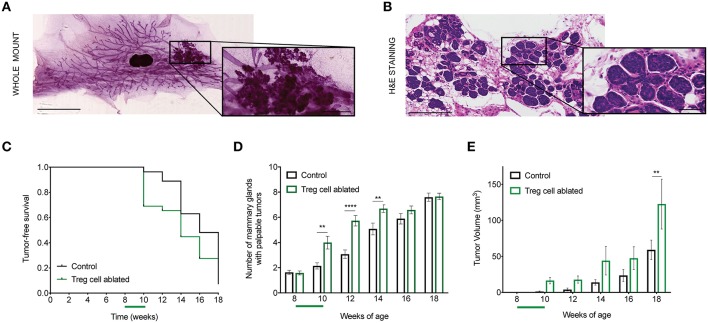
Treg cell ablation accelerates primary tumor growth. **(A,B)** Representative mammary gland whole mount **(A)** and section stained with H&E **(B)** from 8-week-old MMTV-PyMT mouse. Amplified region depicts tumor area **(A)** and hyperplasia/adenoma pre-invasive stage **(B)**, respectively. Scale bars represent 5 mm and 1 mm (lower and higher magnification, respectively; whole mount) as well as 250 and 50 μm (lower and higher magnification, respectively; H&E stain). **(C)** Tumor-free survival curve **(D)**, number of mammary glands with palpable tumors, and **(E)** tumor growth kinetics in Treg cell-ablated mice compared with control mice (*n* = 30–33 mice). ^**^p = 0.006; ^****^p < 0.0001 **(D)** and ^**^p = 0.0075 **(E)**. Values are expressed as mean ± SEM and *p-values* were calculated using two-way ANOVA, followed by Bonferroni's *post-hoc* test. Green bar on the x axis indicates period of Treg cell ablation. Data were pooled from two independent experiments.

In order to investigate the potential role of regulatory T (Treg) cells in the transition from hyperplastic, benign lesions to cancerous lesions, we utilized the polyoma middle-T-driven model of murine breast carcinogenesis (*MMTV-PyMT*) in the C57BL/6 background (14) crossed to *Foxp3*^*DTR*^ mice (15) that we have previously generated (10), to allow for the specific and efficient ablation of Treg cells. This transgenic breast cancer model has been molecularly characterized as clustering with the luminal type of human breast cancer (16), and shows well-defined stages of tumor development that progress through hyperplasia/adenoma, early carcinoma and late carcinoma (Supplementary Figure 1) (12). First, we performed mammary gland whole mounts (Figure 1A) and histological examination of hematoxylin and eosin stained sections (Figure 1B) to identify the time point at which hyperplasia/adenoma was mostly found. Consistent with previous reports (17, 18), we identified the 8-week-old mammary gland as the one showing consistent benign lesions. At this early stage, frequency of Treg cells was similar to the naïve mammary gland (Supplementary Figure 2A). We performed ablation of Foxp3^+^ Treg cells at this time point by intravenous injection of diphtheria toxin (DT) at a 25 μg/kg dose on days 0, 2, 4, as depicted (Supplementary Figure 2B). Using this ablation schedule, Treg cells are almost completely lost from the peripheral blood lymphocyte population by 24 h after the first injection, and remain at low levels for about a week after that, followed by a slow recovery of initial circulating levels by 2 weeks after initial treatment (Supplementary Figure 2C). Importantly, analysis of the mammary gland 14 days after the first DT injection showed significantly reduced Treg cells, suggesting that tissue-specific ablation is more stable than in the periphery (Supplementary Figure 2D). Of note, with this schedule, there is low mouse morbidity, and after treatment is stopped, animals recover.

We then compared the effects of this treatment on the MMTV-PyMT mice. First, we evaluated tumor-free survival over time, and only found a slight acceleration of tumor initiation upon DT treatment (Figure 1C). However, when we counted the number of mammary glands developing tumors in each group, we found more tumor-bearing glands in the DT-treated mice (Figure 1D). There were no apparent differences in the pattern of tumor location between the two groups. Moreover, based on calculated tumor volume, there was a significant increase in the size of the tumors in those mice that underwent Treg cell ablation (Figure 1E). These results suggest that Treg cell presence in the breast environment represents a constrain on invasive progression during early stages of breast cancer.

### Treg Cell Ablation Results in Progression to Early Invasive Carcinoma

The rapid growth of tumors in the 8-week-old, Treg cell ablated mice led us to examine their histopathological characteristics. To that end, we collected the abdominal mammary glands from both groups of mice 14 days after the first DT injection (10-week-old mice), and performed whole mounts followed by sectioning and H&E staining.

**Figure 2:**
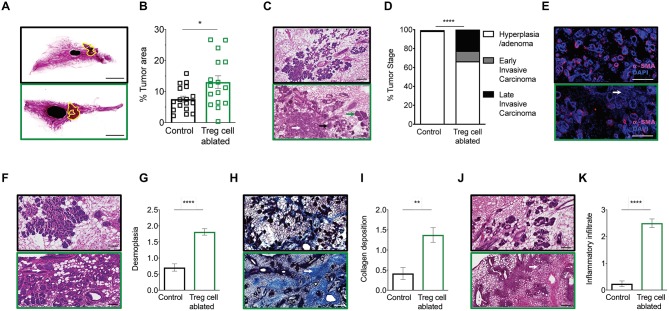
Treg cell ablation results in advanced tumor staging. **(A,B)** 10-week-old mammary gland whole mount representative images **(A)** and quantification **(B)** of the percentage of tumor area of control (black) and Treg cell-ablated (green) mice (*n* = 16–17 individual mammary glands). Scale bars represent 5 mm, ^*^p = 0.0405 calculated by Mann-Whitney test. **(C,D)** Representative image **(C)** and quantification **(D)** of tumor stage. Arrows indicate examples of various tumor stages: hyperplasia/adenoma (green arrow), early invasive carcinoma (black arrow), and late invasive carcinoma (white arrow) **(E)** α-SMA myoepithelial staining confirming disruption of myoepithelial layer **(F,G)**, representative image **(F)** and quantification **(G)** of desmoplasia; **(H,I)** representative image of Masson trichome staining **(H)** and quantification **(I)** of collagen deposition; and **(J,K)** representative image **(J)** and quantification **(K)** of inflammatory infiltration. **(E,F,H,J)** White arrows indicate examples of histological observation. Values are expressed as mean ± SEM. Tumor staging was compared by two-way ANOVA, ^****^p < 0.0001; other comparisons were done by Mann-Whitney test. ^****^p < 0.0001 and ^**^p = 0.0021. Data were pooled from four independent experiments. **(C,E,F,H,J)** Scale bars represent 250 μm.

Corresponding with the increased tumor volumes measured in Treg cell ablated mice at similar temporal points, evaluation of the abdominal mammary gland whole mounts demonstrated significantly greater tumor areas within the gland in Treg cell ablated mice compared to control (Figures 2A,B). Examination of the histological sections by a blinded breast pathologist at 10 weeks, revealed that Treg cell ablation led to more advanced tumors, with increased proportion of early invasive carcinomas (Figures 2C,D). The presence of invasion was confirmed by immunofluorescent staining of alpha-smooth muscle actin (α-sma), which showed an evident disruption of the myoepithelial cell layer (Figure 2E). In addition, histological sections from Treg cell ablated tumors displayed a much higher degree of reactive stroma, characterized by increased desmoplasia (Figures 2F,G), collagen deposition (Figures 2H,I), and intra-tumor inflammatory infiltration (Figures 2J,K).

Together, these observations confirm that Treg cell ablation during non-invasive breast cancer stage induces histological changes associated with progression of the disease to early invasive carcinoma.

### Ablation of Treg Cells Results in Expansion of the Mammary Cancer Stem/Progenitor Cell Pool

**Figure 3:**
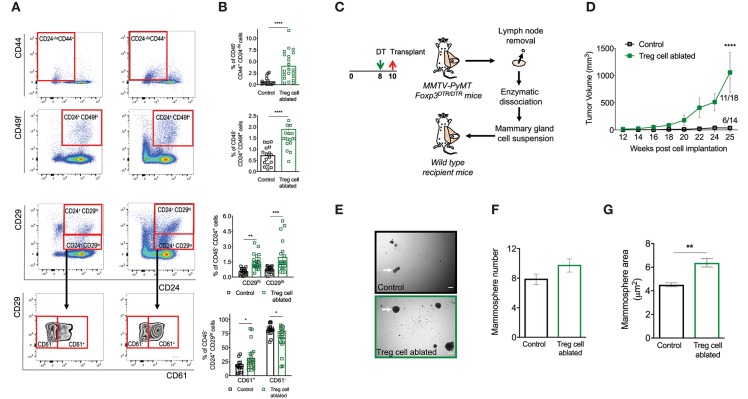
Ablation of Treg cells results in expansion of the mammary cancer stem/progenitor cell pool. **(A,B)** Representative flow cytometric plots **(A)** and quantification **(B)** of stem cell like-populations (CD24^−/lo^ CD44^+^, CD24^+^ CD49f^+^, CD24^+^ CD29^hi^) and luminal progenitor-enriched population (CD24^+^ CD29^lo^) of Treg cell-ablated and control mice. Gating on the luminal progenitor-enriched population, we compared immature (CD61^+^) and differentiated luminal progenitors (CD61^−^) between both groups (bottom). Indicated cell populations highlighted with red frames. Values are expressed as mean ± SEM, control *n* = 11, Treg cell ablated *n* = 12, ^****^p < 0.0001, and was calculated by Mann-Whitney test. ^**^p = 0.0045; ^***^p = 0.0003; ^*^p = 0.0237; ^*^p = 0.0219 was calculated by two-way ANOVA, followed by Bonferroni's *post-hoc* test. Data were pooled from four independent experiments. **(C,D)** Schematics **(C)** and tumor growth kinetics **(D)** of mice orthotopically transplanted with dissociated mammary epithelial cells. Values expressed as mean ± SEM. ^****^p < 0.0001 by two-way ANOVA, followed by Bonferroni's *post-hoc* test **(D)**. **(E–G)** *In vitro* mammosphere forming capacity of CD45 depleted-mammary cells. Representative images **(E)**, mammosphere number **(F)** and area **(G)** of control (black) and Treg cell-ablated (green) conditions (*n* = 16 and *n* = 13, respectively). Arrow depicts a representative mammosphere in each image. Scale bar represents 200 μm. Values are expressed as mean ± SEM. ^**^p = 0.0069 was calculated by Mann-Whitney test. Data were pooled from five independent experiments.

Treg cells have recently been recognized as critical regulators of stem cell homeostasis (19–21). Moreover, tumor initiation, progression, spread and resistance to therapy is dependent on the activity of a small population of cells with the ability to self-renew (22). Given the increased incidence and aggressiveness of tumors in mice that had been depleted of Treg cells, we wondered if this was due, at least in part, to the modification of the cancer stem cell niche. To explore the effect of Treg cell ablation on mammary cancer stem cell pool, we utilized previously defined flow cytometric staining to delineate mouse mammary cancer stem/progenitor cell population (13, 23–26). When we compared dissociated mammary glands from control and Treg cell-ablated mice, we found that treatment resulted in a significant expansion of CD45^−^ CD24^−/lo^ CD44^+^ and CD45^−^ CD24^+^ CD49f^+^ stem cell like-populations as well as, CD45^−^ CD24^+^ CD29^hi^ basal stem cell- and CD45^−^ CD24^+^ CD29^lo^ luminal progenitor-enriched population (Figures 3A,B). Furthermore, we observed that the increase of the luminal progenitor-enriched population was due to an expansion of an immature luminal progenitors (CD45^−^ CD24^+^ CD29^lo^ CD61^+^) over differentiated ones (CD45^−^ CD24^+^ CD29^lo^ CD61^−^) (Figures 3A,B bottom row).

To interrogate these differences functionally, we evaluated the tumor initiating capacity of the mammary cells through a transplantation experiment. We treated mice with DT as before, dissected the mammary glands at 10 weeks of age, and prepared single cell suspensions. We orthotopically transplanted 325,000 dissociated cells into a naïve host, and evaluated tumor appearance and volume over time. Consistent with our previous observations, tumors manifested earlier, and incidence was higher from the suspensions prepared from DT-treated mice (11 out of 18 transplants vs. 6 out of 14 from control mice) (Figures 3C,D).

Finally, in order to evaluate cancer stem cell activity more directly, we isolated CD45-negative mammary cells from Treg cell ablated and control mice, and seeded them in non-adherent, mammosphere-forming conditions during 7 days. We found that mammary gland cells from DT-treated mice grew a similar number, but bigger mammospheres at the end of the assay (Figures 3E–G).

Combined, these experiments highlight an important role for Treg cells in the homeostasis of the breast stem cell-like population.

### Treg Cell Ablation Results in an Immune Microenvironment Associated With Tumor Progression

**Figure 4:**
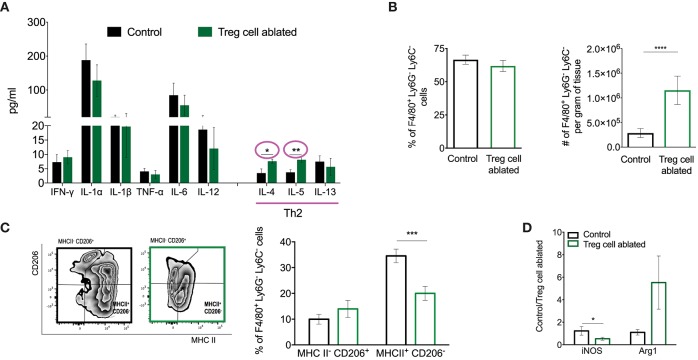
Treg cell ablation results in an immune microenvironment associated with tumor progression. **(A)** Multiplex analysis of shown cytokines. Values are expressed as mean ± SEM, *n* = 3 mice per group. ^*^p = 0.0278 and ^**^p = 0.0082 were calculated by unpaired *t*-test. **(B)** Percentage (left) and absolute number (right) of tumor associated macrophages (CD11b^+^ LY6C^−^ LY6G^−^ F4/80^+^). ^****^p < 0.0001 was calculated by Mann–Whitney test. **(C)** Representative flow cytometric plot (left) and quantification (right) of TAM (Ly6G^−^ Ly6C^−^ F4/80^+^) cell polarization as relative amounts of MHCII^−^ CD206^+^ and MHCII^+^ CD206^−^ cells. ^***^p < 0.0007 was calculated by two-way ANOVA, followed by Bonferroni's *post-hoc* test. **(D)** Mammary gland relative expression of iNOS and Arg-1 (M1 and M2 markers, respectively) quantified by qPCR. Values are expressed as mean ± SEM and were normalized by F4/80 and beta-actin levels. *n* = 5–6, ^*^p = 0.0479 was calculated by unpaired *t*-test. Data were pooled from two independent experiments.

In order to shed light into the mechanisms by which Treg cell ablation promotes progression of non-invasive into invasive breast cancer, we evaluated changes in the cytokine milieu of the mammary gland upon DT treatment. For the most part, no changes were observed in TH1-related cytokines such as IFNγ, IL-12, or TNFα. However, we observed significant elevation of the TH2 cytokines IL-4 and IL-5 (Figure 4A). Furthermore, we observed significantly higher number of F4/80^+^ macrophages in the mammary gland tissue of Treg cell-ablated mice (Figure 4B). Alternative activation of macrophages mediated primarily by IL-4 leads to an array of pro-tumorigenic functions that promote tumor progression, dissemination, and inhibit response to therapy (27). To address this, we looked at the polarization status of F4/80^+^ cells in the mammary gland by flow cytometry, evaluating the CD206/MHCII cell ratio. Consistent with the increase in TH2 cytokines, we observed a significant reduction in the MHCII^+^CD206^−^ macrophage subset, with a slight increase in the CD206^+^MHCII^−^ population (Figure 4C). Furthermore, we performed semi-quantitative real time PCR on RNA extracted from the mammary glands, and detected significantly less iNOS and more Arg1 in tissues (Figure 4D), after normalization for the macrophage marker F4/80 and the housekeeping gene beta-actin. Altogether, our observations suggest that Treg cells in the early breast cancer microenvironment function to prevent the establishment of a pro-tumorigenic microenvironment, which results in delayed tumor invasion.

## Discussion

The early events leading to progression of *in situ* breast lesions to invasive cancer are poorly understood (7). While all patients with DCIS are heavily treated with surgery and radiation at least due to the lack of biomarkers, for most of them this results in unnecessary morbidities and side effects (1). Moreover, early intervention with targeted therapies is not possible despite the fact that a subset of DCIS patients will go on to develop invasive cancer (4). Thus, understanding the cellular or molecular mechanisms that govern the transition from non-invasive to invasive cancer is critical.

Breast cancer accumulates Foxp3^+^ Treg cells upon tumor progression, and we have demonstrated that transient ablation of Treg cells in established, highly immuno-suppressive breast tumors results in a significant increase in anti-tumor immunity in primary and metastatic tumors (10). In this context, while cytotoxic T and NK cell activity is dispensable for the antitumor effect, IFNγ-dependent reprogramming of the tumor microenvironment is required (10). In contrast, intraductal immune cell accumulation is rarely detected in early DCIS lesions (28), and Treg cell frequency in normal and neoplastic 8-weeks mammary gland is similar, suggesting a microenvironment more similar to the normal gland. In this study, we found that transient Treg cell ablation at this pre-invasive breast tumor stage accelerates the rate of tumor progression to invasive cancer, increasing the number of mammary glands harboring tumors and promoting the development of early invasive carcinoma. In addition, Treg cell ablation heightened mammary reactive stroma, characterized by a higher desmoplasia and collagen deposition. In line with our observations, this stromal change has been associated with the activation of angiogenic programs, recruitment of inflammatory cells, invasive phenotype, and metastatic progression (29).

It is now well-established that Treg cells play critical roles in maintaining non-lymphoid tissue homeostasis (30–32). More recently, a relationship between Treg cells and tissue-specific stem cells has been identified. In the bone marrow, Treg cells create an immune-privileged site enabling allo-hematopoietic stem/progenitor cell persistence and quiescence (19, 20). In addition, skin Treg cells play a major role in hair follicle biology by promoting the function of hair follicle stem cells (21). Cancer stem cells are required for the initiation, progression, metastatic dissemination and response to therapy in breast cancers (33, 34). Here, we describe a previously unrecognized effect of Treg cells on mammary cancer stem/progenitor cells during the early stages of tumorigenesis. Specifically, Treg cell ablation induced expansion of CD45^−^ CD24^−/lo^ CD44^+^, CD45^−^ CD24^+^ CD49f^+^, and CD45^−^ CD24^+^ CD29^hi^ stem cell like-populations, as well as an immature luminal progenitor-enriched population (CD45^−^ CD24^+^ CD29^lo^ CD61^+^). The murine CD44^+^ CD24^−^ cancer stem cell population found in the primary tumors of MMTV-PyMT transgenic mice exhibits functional characteristics of human breast cancer stem cells (23), which highlights the clinical impact of our finding. Our data suggest that Treg cells negatively regulate the early cancer stem cell niche. Supporting this, we demonstrated that dissociated mammary gland from Treg cell ablated mice progressed into tumors faster and with increased penetrance after transplantation into naïve hosts. Additionally, mammospheres from Treg cell ablated mice were significantly larger when cultured under non-adherent conditions. Whether this is a direct effect of the Treg cell interaction with the stem cell niche or an indirect effect due to changes within the tumor microenvironment that occur after Treg cell depletion remains to be investigated. Furthermore, our unpublished observations suggest that similar expansion of normal mammary gland stem cell is observed when Treg cells are ablated in naïve mammary gland (data not shown).

Lastly, we found dysregulated amounts of IL-4 and IL-5 cytokines, and a concomitant increase in the number of tumor associated-macrophages (TAMs). Whether the increase in macrophages is due to expansion of tissue-resident populations or recruitment of inflammatory monocytes remains to be determined. Consistent with the increase in TH2-type cytokines, we observed increased frequencies of alternatively activated TAMs, as defined by their expression of CD206 and MHCII. Furthermore, semi-quantitative PCR to detect macrophage effectors Arg1 and iNOS from the mammary gland tissue after Treg cell ablation suggested qualitative changes in the macrophage infiltrate. Specifically, we observed lower levels of iNOS (classical activation marker), and higher levels of Arg1 (alternative activation marker). TH2 cytokines such as IL-4 have been shown to induced tumorigenic properties in TAMs (35), facilitating invasion and metastasis by regulating their phenotype and function (36), such as the production of cathepsins B and S (37). These results suggest that Treg cells regulate the immune environment of non-invasive breast cancer at least in part by their effects on mammary gland macrophages. Future studies utilizing genetic or chemical deletion of macrophages will be necessary to evaluate this possibility. It is interesting to note that the changes observed upon Treg cell ablation in the hyperplastic mammary gland are similar to those taking place during the involution of the lactating mammary gland, a state that has been mechanistically linked to the increased chance of metastatic recurrence observed in pregnancy-associated breast cancer (38).

In summary, our study demonstrates that Treg cells prevent the transition of pre-invasive to invasive breast cancer by selectively suppressing pro-tumorigenic TH2 responses and restraining the cancer stem cell pool. Ongoing and future studies will shed light into the cellular mechanisms underlying this observation. Furthermore, validating whether increased numbers of Treg cells present within early *in situ* breast lesions associates with a more favorable outcome could justify future studies to investigate the potential of Treg cells, macrophage infiltrates, and stem cell profiles as biomarkers that accurately enable identification of the DCIS patients that will most likely benefit from receiving radiation therapy and surgery. These studies should help to contribute to the development of paradigm shifting standard of care treatment for DCIS patients.

## Data Availability

The raw data supporting the conclusions of this manuscript will be made available by the authors, without undue reservation, to any qualified researcher.

## Author Contributions

LM and PB designed the study, analyzed the data, and wrote the manuscript. VR performed histopathological analysis of mammary glands. NC and WD assisted with flow cytometry studies. MI supervised histological analysis. MR contributed to the interpretation of the studies. All authors edited the manuscript.

### Conflict of Interest Statement

The authors declare that the research was conducted in the absence of any commercial or financial relationships that could be construed as a potential conflict of interest.

## Supplementary Material

Supplementary Figure 1 Tumor staging **(A)** representative picture of an H&E stained MMTV-PyMT mammary gland exemplifying the different tumor stages **(A)** hyperplasia/adenoma **(B)**, early invasive carcinoma, and **(C)** late invasive carcinoma. Scale bars represent 500 and 100 μm (lower and higher magnifications, respectively).

Supplementary Figure 2 Treg cell frequencies and ablation efficiency. **(A)** Frequency of Treg cells (CD4^+^ Foxp3^+^) in the mammary gland of naïve and MMTV-PyMT mice at 8 weeks of age. Representative of two independent experiments with similar results. **(B)** Schematic of Treg cell ablation. Foxp3^+^ Treg cells were ablated by intravenous injection of DT on days 0, 2, and 4 in 8-week-old mice. **(C)** Kinetics of Treg cell frequency in peripheral blood in control (black) and DT-treated (green) mice (*n* = 4, respectively). Values are presented as percentage of day 0. **(D)** Frequency of Treg cells (CD4^+^ Foxp3^+^) in the mammary gland of control and DT-treated MMTV-PyMT mice at 10 weeks of age (2 weeks after initial treatment). ^*^*p* < 0.05 by two-tailed unpaired *t*-test. Data were pooled from three independent experiments.
