# Supplementary figures and images for: Regulatory T Cells Control the Switch From in situ to Invasive Breast Cancer
Source: Front Immunol. 2019 Aug 29;10:1942. doi: 10.3389/fimmu.2019.01942 (PMC6727150; doi:10.3389/fimmu.2019.01942)

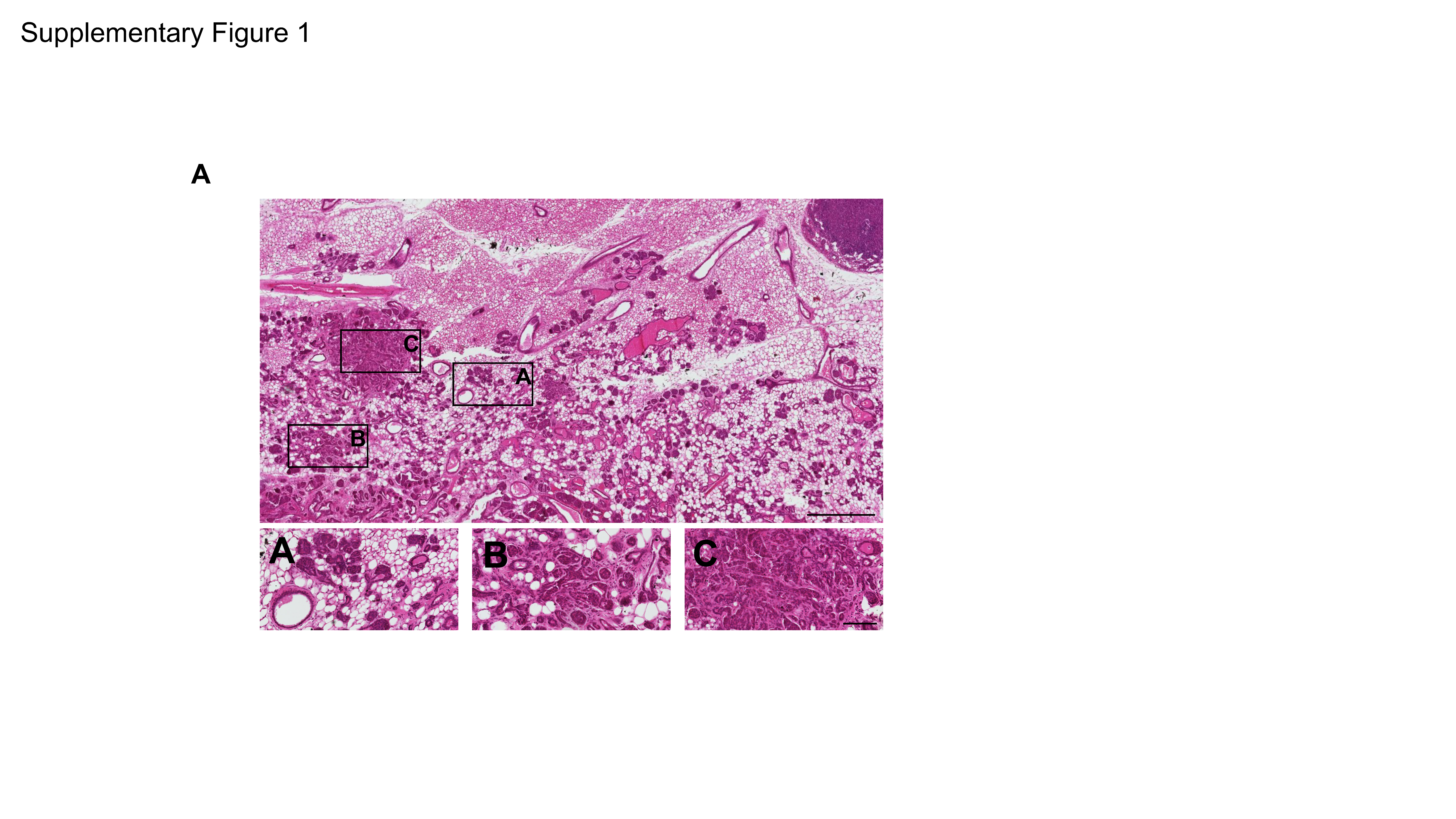

Supplement: Supplementary Figure 1 — Tumor staging (A) representative picture of an H&E stained MMTV-PyMT mammary gland exemplifying the different tumor stages (A) hyperplasia/adenoma (B), early invasive carcinoma, and (C) late invasive carcinoma. Scale bars represent 500 and 100 μm (lower and higher magnifications, respectively). [file Image_1.TIFF]

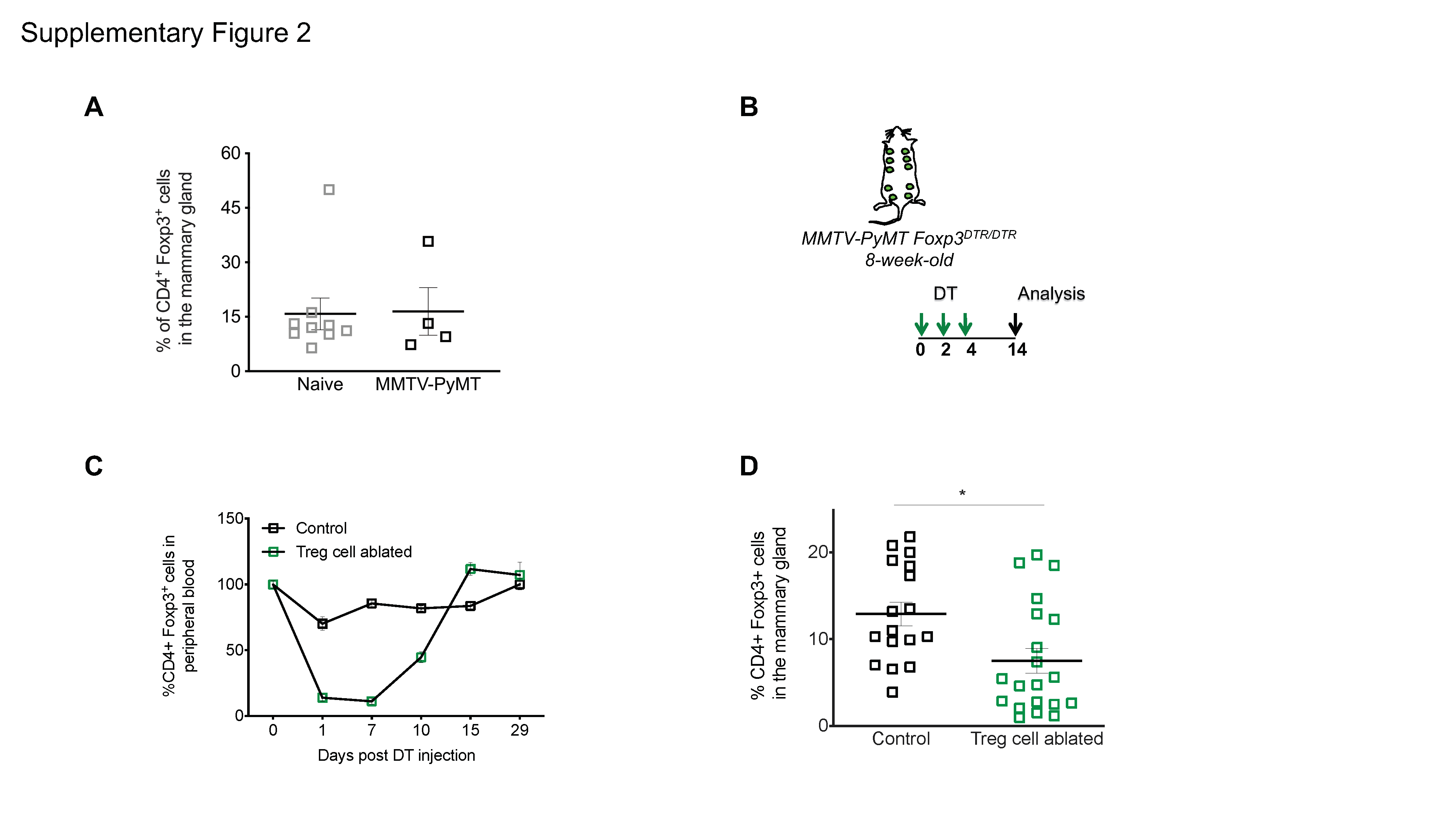

Supplement: Supplementary Figure 2 — Treg cell frequencies and ablation efficiency. (A) Frequency of Treg cells (CD4+ Foxp3+) in the mammary gland of naïve and MMTV-PyMT mice at 8 weeks of age. Representative of two independent experiments with similar results. (B) Schematic of Treg cell ablation. Foxp3+ Treg cells were ablated by intravenous injection of DT on days 0, 2, and 4 in 8-week-old mice. (C) Kinetics of Treg cell frequency in peripheral blood in control (black) and DT-treated (green) mice (n = 4, respectively). Values are presented as percentage of day 0. (D) Frequency of Treg cells (CD4+ Foxp3+) in the mammary gland of control and DT-treated MMTV-PyMT mice at 10 weeks of age (2 weeks after initial treatment). *p < 0.05 by two-tailed unpaired t-test. Data were pooled from three independent experiments. [file Image_2.TIFF]
